# Surgical Treatment for Giant Multiple Coronary Artery Aneurysms Caused by an IgG4-Related Disease

**DOI:** 10.7759/cureus.60115

**Published:** 2024-05-11

**Authors:** Takura Taguchi, Hiroyuki Nishi, Mutsunori Kitahara, Yukie Shirasaki, Masao Yoshitatsu

**Affiliations:** 1 Department of Cardiovascular Surgery, National Hospital Organization, Osaka National Hospital, Osaka, JPN

**Keywords:** resection of aneurysm, coronary artery bypass grafting(cabg), cardiovascular surgery, igg4-related disease, coronary artery aneurysm

## Abstract

Coronary artery aneurysms (CAAs) due to an immunoglobulin G4 (IgG4)-related disease (IgG4-RD) are relatively rare, and there is no consensus on the choice of treatment method. In the present study, we report the results of the surgical treatment for multiple giant CAAs caused by IgG4-RD. A 71-year-old man was diagnosed with severe aortic regurgitation and CAAs. A blood test showed high IgG4 levels, and computed tomography revealed four giant coronary artery aneurysms: two in the right coronary artery (RCA) (proximal RCA and posterior descending artery (PDA)), one in the left anterior descending (LAD), and one in the diagonal branch (Dx). We planned aortic valve replacement, coronary aneurysm resection, and coronary artery bypass grafting (CABG). After finishing aortic valve replacement, the CAAs in proximal RCA, LAD, and Dx were resected. The proximal and distal tracts of the aneurysm were closed with a pericardial bovine patch and ligation. However, since the distal PDA was too calcified to be anastomosed, and the PDA aneurysm was smaller than the others, it was decided to leave the PDA aneurysm. The anastomoses of SVG-RCA and Dx, as well as the left internal thoracic artery to LAD, were performed. Histopathological examination of the aneurysm wall showed a high IgG4-positive cell/IgG-positive cell ratio, and a diagnosis of IgG4-RD was made. In the treatment of CAAs due to IgG4-RD, it is essential to select a procedure that takes into account the size, location, and nature of the aneurysm, and comorbidities. To ensure resection of the aneurysm and blockade of blood flow, closure of the inflow and outflow tracts with a pericardial bovine patch and CABG are effective.

## Introduction

Immunoglobulin G4 (IgG4)-related disease (IgG4-RD) is an immune-mediated condition characterized by fibroinflammatory lesions, a dense lymphoplasmacytic infiltrate rich in IgG4-positive plasma cells, and storiform fibrosis that can occur at nearly any anatomic site [1,2]. The clinical manifestations when the cardiovascular system is involved could include aortitis/arteritis and inflammatory aneurysms [3]. A coronary artery aneurysm (CAA) is defined as a focal dilation of more than 1.5 times the adjacent normal area, with the risk of rupture or possible myocardial infarction due to a thrombus. Therefore, medical treatment, such as antiplatelets or anticoagulation, percutaneous coronary intervention (PCI), and surgery, are recommended. However, without extensive data, there is no consistent evidence, and IgG4-RD is particularly rarely reported [4]. Herein, we report a successful surgical treatment for multiple giant coronary artery aneurysms (CAAs) caused by IgG4-RD.

## Case presentation

A 71-year-old man with no medical history was transferred to our hospital due to impaired consciousness. Head computed tomography (CT) showed no abnormal findings. A physical examination revealed a diastolic murmur. Chest radiography showed cardiomegaly (cardiothoracic ratio of 65%). Electrocardiography showed sinus rhythm, incomplete left branch block, and no significant ST-T change. Echocardiography revealed severe aortic valve regurgitation (AR) with tricuspid of the aortic valve, dilated left ventricle (end-diastolic dimension/end-systolic dimension = 66/56 mm), decreased left ventricular ejection fraction (EF = 31%), and multiple giant CAAs. He was diagnosed with impaired consciousness due to heart failure, and his consciousness improved after treatment. Then, he was referred to our department for surgical treatment.

**Figure 1:**
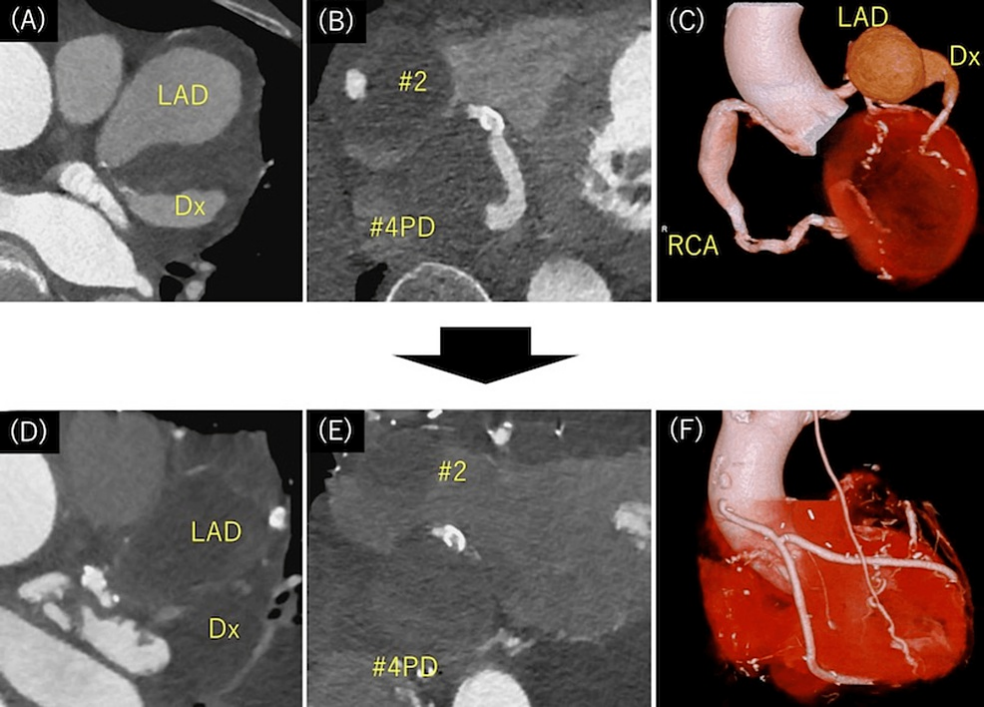
Preoperative (A-C) and postoperative (D-F) cardiac computed tomography (A, B) Preoperative horizontal view. (C) 3D image; LAD aneurysm was close to the LMT. (D, E) Postoperative horizontal view; there was no blood flow in all CAAs. (F) All grafts were patent. LMT: left main trunk; LAD: left anterior descending; Dx: diagonal branch; PD: posterior descending; RCA: right coronary artery; CAA: coronary artery aneurysm

Upon admission, laboratory test results showed the following: leukocytes 4300/μL, C-reactive protein 0.5 mg/dL, brain natriuretic hormone 481.6 pg/mL, IgG 1845 mg/dL, and IgG4 282 mg/dL. Enhanced coronary CT revealed multiple giant CAAs: proximal right coronary artery (RCA) (50×56 mm), posterior descending artery (PDA) (25×30 mm) (Figure 1B), left anterior descending (LAD) (56×62 mm), diagonal branch (Dx) (26×32 mm) (Figure 1A). LAD aneurysm was close to the left main trunk (LMT) (Figure 1A, 1C). Coronary angiogram showed contrast agent stagnation in CAAs of RCA and LAD.

**Figure 2:**
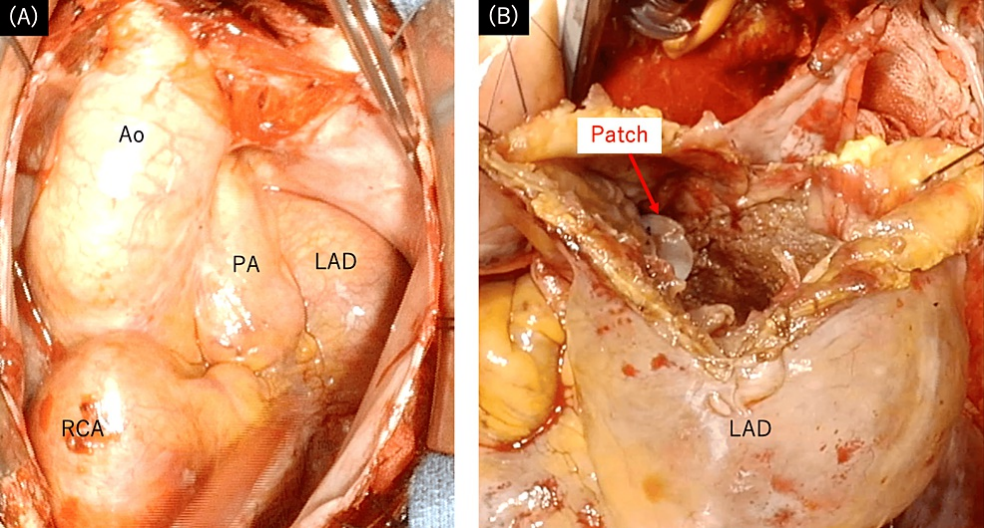
Intraoperative images (A) Giant coronary artery aneurysms of the RCA and LAD have been exposed. (B) The proximal side of the LAD aneurysm was closed using the bovine pericardial patch. Ao: aorta; PA: pulmonary artery; LAD: left anterior descending; RCA: right coronary artery

The surgical strategy of CAAs was coronary artery bypass graft (CABG) to a distal segment from the CAAs, aneurysm resection, and closure of inflow and outflow sites of CAAs. The patient underwent aortic valve replacement (AVR), CABG, and CAAs resection. We performed a median sternotomy, noted giant CAAs of RCA and LAD (Figure 2A), and harvested the left internal thoracic artery (LITA) and great saphenous vein (SVG). Cardiopulmonary bypass (CPB) was established with bicaval drainage and perfusion to the ascending aorta. Then, AVR (Epic 23mm, St Jude Medical, Minnesota, USA) was done after cardiac arrest. Since the distal RCA was so calcified that anastomosis was impossible and the PDA aneurysm was small, we decided not to resect the PDA aneurysm. When the CAAs (proximal RCA, LAD, and Dx) were incised, a lot of thrombi were found in the CAAs, and each inflow and outflow site was closed. The inflow site of LAD and proximal RCA was closed with the bovine pericardial patch (Figure 2B), and the other sites were ligated. Then, an aorto-coronary bypass to PDA and Dx was performed using SVG. After LITA was anastomosed to distal LAD, the cross-clamp was released. After an intra-aortic balloon pump was inserted, the weaning from CPB was uneventful.

**Figure 3:**
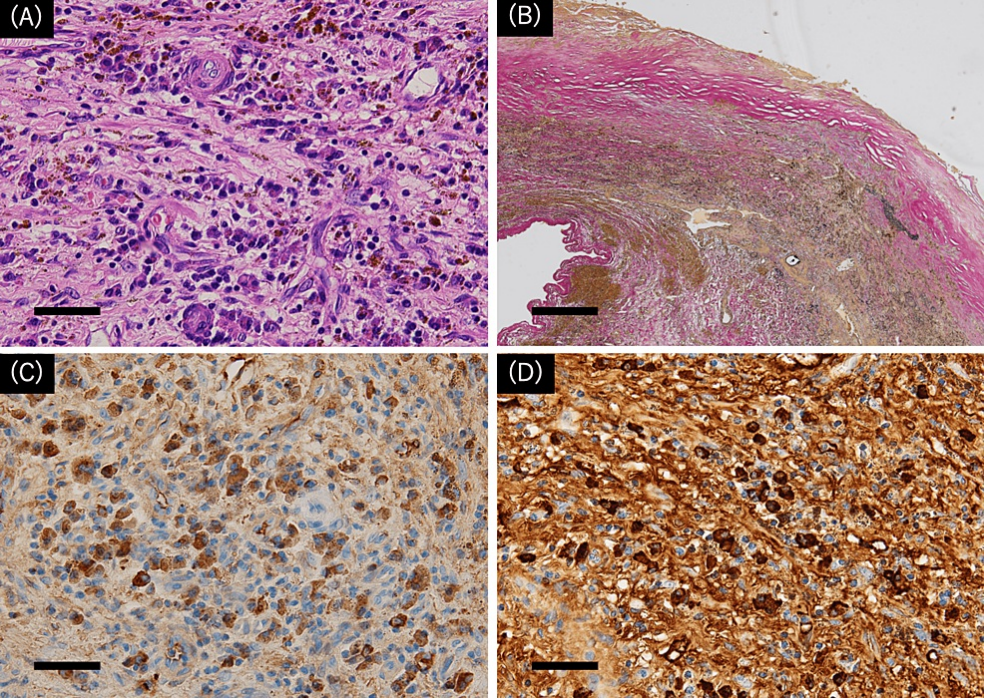
Postoperative pathological examination of coronary artery aneurysms (A) H&E-stained section, x400. Scale bar, 50 μm. Marked infiltration of plasma cells and small lymphocytes is identified in media to the adventitia of the coronary artery. Hemosiderin-laden macrophages are also identified. (B) Elastica van Gieson (EVG)-stained section showed evident thinning of media and fibrous thickening of intima with mural thrombus. Scale bar, 500 μm. (C) IgG4 immuno-stained section, x400. Scale bar, 50 μm. More than 40 IgG4-positive plasma cells are counted in a high-power field. (D) IgG immuno-stained section, x400. Scale bar, 50 μm. Less than 100 IgG-positive plasma cells are counted in a high-power field, which turned out to be a positivity ratio between IgG4 and IgG (IgG4/ IgG) > 40%. Ig: immunoglobulin

Although the patient required a tracheotomy and implantation of a pacemaker due to sick sinus syndrome, the postoperative course was stable without cardiac failure. Postoperative CT showed no blood flow in all CAAs and patency of graft (Figures 1D-1F), and echocardiography revealed no abnormal findings with no change in EF. Antiplatelet and anticoagulant drugs were used for graft patency and to prevent thrombus formation in the coronary artery. Postoperative pathological examination of CAA revealed infiltration of lymphocytes, plasma cells, IgG-positive plasmacytes, and IgG4-positive plasmacytes and fibrosis (Figure 3). The plasmacyte IgG4/IgG ratio was 66%.

## Discussion

CAAs develop due to various causes, including arteriosclerosis, arteritis, infection, trauma, and some cases, such as this case, IgG4-RD. The right coronary artery is usually the most affected (40%), followed by the left anterior descending (32%), and the left main is the least affected artery (3.5%) [1]. IgG4-RD is a newly recognized fibroinflammatory condition characterized by tumefactive lesions, a dense lymphoplasmacytic infiltrate rich in IgG4-positive plasma cells, and storiform fibrosis [2]. IgG4-RD occurs primarily in middle-aged and older men and causes CAA and other aneurysms, which may require surgical treatment. In this case, the diagnosis of IgG4-related disease was made based on the presence of a giant CAA on CT, high levels of IgG4 (282 mg/dL > 135 mg/dL) in the blood, and infiltration of lymphocytes, plasma cells, and IgG4-positive plasma (IgG4/IgG ratio: 66% > 40%) and fibrosis on pathology [3].

The treatment for CAAs includes medical management, PCI, and surgical treatment, with surgical treatment recommended for giant aneurysms (>20 mm), multiple aneurysms, and an aneurysm of LMT [4]. Although there are few reports of CAA caused by IgG4-RD, much less multiple giant CAAs, rupture of giant CAAs as large as 5 cm, and sudden death from myocardial infarction due to thrombus have been reported [5,6]. In this case, the patient had CAAs over 5 cm in size in the RCA and LAD, which were at risk of rupture and cardiac dilation with severe AR, which led to the decision for surgical treatment.

Surgical treatment for CAA in a patient with IgG4-RD is rare, with only four cases reported thus far (Table 1). The cases were single and giant CAAs, and the origin of the CAA was the RCA. There were no case reports of surgical treatment for giant and multiple CAAs due to IgG4-RD, such as the present case. Although CABG was performed in all cases, with regard to the treatment for the CAA, some cases were direct suture closure of inflow and outflow sides [7,8], and others were coil embolized [9]. In an aneurysm of LAD close to LMT, as in this case, patch closure of inflow and outflow tracts is effective, given the risk of stenosis of LMT.

**Table 1:** Summary of the case reports of surgical treatment for coronary artery aneurysm in patients with IgG4-related disease (1) Direct suture closure was performed at the inflow and outflow sites of the aneurysm. (2) Patch closure was performed with the bovine pericardial patch at the inflow sites of LAD and RCA aneurysm. Direct suture closure was performed at the other sites of the aneurysm. CAA: coronary artery aneurysm; RCA: right coronary artery; LCx: left circumflex artery; LAD: left anterior descending artery; Dx: diagonal artery; CABG: coronary artery bypass grafting; Ig: immunoglobulin

Author	Age (years)	Sex	Origin of CAA	Size (mm)	Steroid	Reason for steroid	CABG	How to close the CAA
Pota et al. 2021 [5]	65	M	RCA	53×54	Pre & Post	recurrence new CAA	Yes	none noted
Matsuyama et al. 2020 [7]	74	M	RCA	85×65	Pre & Post	inflammatory AAA	Yes	direct suture closure ^(1)^
Ikutomi et al. 2011 [8]	75	M	RCA	40×48	Pre & Post	pancreatitis parotitis	Yes	direct suture closure^(1)^
Kamikawa et al. 2021 [9]	62	M	LCx	38	Pre & Post	pancreatitis, nephritis	Yes	endovascular embolization
Present case	71	M	RCA, LAD, Dx	50×56, 56×62, 26×32	none	none	Yes	direct suture closure patch closure^(2)^

In these cases, IgG4-RD was initially diagnosed, and steroids were used. It reduces inflammation by suppressing lymphocyte activation, which is expected to prevent the further development of inflammatory lesions, including aortitis/arteritis and aneurysm. On the other hand, it is not able to shrink the size of an already-developed aneurysm [10]. In addition, the reduction of a periarterial pseudotumor and thinning of the arterial wall by steroid therapy might increase the risk of aneurysm rupture [11]. Therefore, the decision to use steroids should be based on a combination of these factors and should be done with caution. In this case, steroid was not introduced because there were no other inflammatory findings. However, since there is a possibility that not only small and medium-sized arteries but also the abdominal aorta may develop aneurysms and inflammatory diseases may develop not only in blood vessels but also in internal organs, we plan to observe inflammatory findings by laboratory data and PET scans, as well as regular systemic examinations, and introduce steroids as necessary. Since CAAs caused by IgG4-RD are rare, and there is still little information available, future follow-up, including this case, is essential.

## Conclusions

We successfully performed surgical treatment of giant multiple CAAs in a patient with IgG4-RD. Giant and multiple IgG4-related CAAs are very rare, and the choice of surgical treatment must consider not only the size, location, and nature of the aneurysm but also comorbidities. Appropriate surgical treatment for aneurysms such as the one in this case is essential, and careful follow-up is necessary in the future.
